# A Novel Method for Estimating the Body Weight and Size of Sows Using 3D Point Cloud

**DOI:** 10.3390/ani16010072

**Published:** 2025-12-26

**Authors:** Hong Zhou, Qiuju Xie, Wenfeng Wang, Jiaming Gu, Honggui Liu, Bin Li, Shuaijun Wu, Fang Zheng

**Affiliations:** 1 College of Electrical and Information, Northeast Agricultural University, Harbin 150030, China; zh_hlj@126.com (H.Z.);; 2 Key Laboratory of Smart Farming Technology for Agricultural Animals, Ministry of Agriculture and Rural Affairs, Wuhan 430000, China; 3 College of Animal Science and Technology, Northeast Agricultural University, Harbin 150030, China; 4 Key Laboratory of Swine Facilities Engineering, Ministry of Agriculture and Rural Affairs, Harbin 150030, China; 5 Intelligent Equipment Research Center, Beijing Academy of Agriculture and Forestry Sciences, Beijing 100097, China; 6 College of Information Science and Technology, Huazhong Agricultural University, Wuhan 430000, China

**Keywords:** precision livestock farming, non-contact measurement, point cloud segmentation, deep learning, DbmoNet

## Abstract

Body weight and size are critical indicators of sow health and reproductive performance. Traditional manual measurement methods are not only time-consuming and labor-intensive but also induce stress in sows. To address these limitations, we propose an innovative method for estimating sow body weight and size using 3D point cloud data. Our method began by obtaining point cloud data from depth images captured by an Intel^®^ RealSense™ D455 camera. First, we used a KPConv segmentation model with a deformable kernel to extract the sow‘s back. The resulting back point cloud then served as the input to a novel dual-branch, multi-output regression model named DbmoNet, which integrates features from both location and feature spaces. We evaluated the method on 2400 samples from three breeds during non-pregnant periods. The KPConv model demonstrated excellent performance, achieving an overall segmentation accuracy (OA) of 99.54%. The proposed DbmoNet model outperformed existing benchmarks, achieving mean absolute percentage errors (MAPEs) of 3.74% for body weight (BW), 3.97% for chest width (CW), 3.33% for hip width (HW), 3.82% for body length (BL), 1.94% for chest height (CH), and 2.43% for hip height (HH). Therefore, this method provides an accurate and efficient tool for non-contact body condition monitoring in intensive sow production.

## Simple Summary

This study proposes a novel non-contact approach for automatically estimating sow body weight and key morphological parameters using 3D point cloud data. Back point clouds, captured by a depth camera, were first segmented using a KPConv model. A newly designed dual-branch regression network (DbmoNet) was then employed to simultaneously predict six metrics: body weight, chest width, hip width, body length, chest height, and hip height. Tested on 2400 samples, the method achieved a mean absolute percentage error of 3.74% for weight estimation, demonstrating its potential as an efficient and reliable solution for precision monitoring in intensive sow production.

## 1. Introduction

Body weight and size are closely related to the health and production performance of live sows [1]. With the development of intensive and precise breeding, monitoring livestock body weight and size has become crucial for optimizing production management and assessing animal welfare [2]. Traditional methods of weighing with a scale and tape measure are time-consuming, labor-intensive, and can cause stress in animals [3]. Machine vision technology offers intuitive and non-contact measurement advantages [4]. In recent years, advancements in sensor and artificial intelligence technology have accelerated research on using machine vision to estimate animal body weight and size [5].

The images obtained by visual sensors can be divided into two-dimensional (2D) images and three-dimensional (3D) images. The technique for determining body weight and size from 2D images is generally to collect 2D RGB images [6,7] or grayscale images [8] of the animal’s back or side, to identify key points of body size measurement through 2D image processing to estimate length, width, and height of body size [9], and to establish a model to correlate body size with body weight to estimate body weight [10]. However, 2D images do not include in-depth information and cannot measure 3D body size parameters involving chest circumference, waist circumference, and hip circumference [11]. At the same time, due to the characteristic of the target being large in the near and small in the far, the image processing process is influenced by the target position and camera perspective [12]. In addition, the complexity of light, lighting, and background can also affect the results [13]. For example, when the light is weak or the sow body and the background color are similar, it will make the segmentation of the target and recognition of feature points more difficult [14].

The 3D images used in current research mainly include depth images and point clouds [15,16]. Body size is generally measured through steps such as point cloud segmentation, point cloud 3D reconstruction, feature point recognition of the depth images and point clouds, and point cloud calculation to further estimate body weight [17]. In some studies, depth images or point clouds of the back of sows can be obtained from a top-view perspective [18,19], and body size feature points can be labeled using point cloud calculations based on the anatomical characteristics of the sow’s back [20,21]. In order to improve the accuracy of body size point recognition, methods for 3D reconstruction of sow bodies are also commonly used [22]. Multiple depth cameras were used to capture images of the back and different sides of the sow body [23]. The sow body and scene were restored through 3D reconstruction, and the background was removed through point cloud segmentation to extract the sow body. After preprocessing processes such as posture normalization, body size feature points were extracted from the reconstructed sow body [24]. Body size features typically include hip width, hip height, chest width, chest height, body length, chest circumference, abdominal circumference, and hip circumference [25]. Some studies extract multiple width features of the back for estimating body weight [26], and the volume of the top view covered by the back depth map is often used as a weight estimation feature [27,28]. In order to facilitate the acquisition of features for weight estimation, Nguyen et al. [29] used the original point cloud as input and extracted 64 potential features using a generative network. Three methods were used to establish models correlating body size features with body weight: Support Vector Regression (SVR), Multilayer Perceptron (MLP), and AdaBoost. To improve the accuracy of body measure point recognition, Hu et al. [30] employed a PointNet++ point cloud segmentation model to divide the pig’s body into various parts, including the ears, head, trunk, limbs, and tail. Within these segmented regions, point cloud processing was utilized to pinpoint key body size measurements, achieving a minimum relative error of 2.28%.

In summary, current methods use image or point cloud processing to identify feature points and extract body size traits. Body weight is then estimated through machine learning algorithms. However, these approaches face several major limitations. First, to ensure data consistency and comparability, sows are often required to maintain a uniform posture during image acquisition. This requirement is difficult to achieve in practical settings, reducing the broad applicability of such methods. Second, the processing pipeline involves multiple complex steps, including 3D reconstruction of point clouds, identification of feature points, and calculation of body size measurements. These operations are computationally intensive and time-consuming. The complexity of feature engineering may also introduce errors, affecting the accuracy and stability of feature extraction. Most importantly, relying on hand-crafted features to build machine learning models may constrain both feature representation and the model’s ability to capture complex patterns, ultimately limiting prediction performance.

In recent years, some studies have explored the feasibility of end-to-end body size and weight estimation using automatic feature extraction. Using depth images as input, body weight and size estimation models such as the Xception model [31], EfficientNets model [32], and Faster RCNN model [33] were established. He et al. [34] preprocessed the point cloud and used distance independent algorithms to map it to 2D grayscale images, establishing a botnet weight estimation model. Both depth and grayscale images are structured data with fixed adjacent positions between them. However, almost all the estimation models used 2D convolution to extract the feature relationship between neighboring positions, thus the extraction accuracy is limited. Point cloud, as an irregular data structure, are able to better characterize 3D space and are therefore a better way to characterize 3D features of sow bodies. Recently, significant advancements have been made in the development of end-to-end algorithms for point cloud segmentation and classification. Extracting features from point clouds is crucial for analyzing 3D scenes. Notably, there are several algorithms that have gained significant attention due to their broad applications and improved results in point cloud classification and segmentation. Some of these algorithms include PointNet++ [35], DGCNN [36], KPConv [37], PointCNN [38], RandLANet [39]. Although existing algorithms have made significant progress in enhancing local feature extraction, they often exhibit limitations due to predefined or fixed receptive fields, and primarily perform neighborhood construction and feature aggregation within a single domain. This often results in an inability to fully capture the multifaceted relationships between points, thereby restricting the representational capacity of the features.

To overcome these limitations, this study aims to develop a novel framework for accurate and robust, non-contact estimation of sow body weight and size from 3D point clouds. The specific objectives are as follows:

1. To establish an effective point cloud segmentation model for extracting the sow‘s back from complex barn backgrounds.
2. To propose a dual-branch network architecture (DbmoNet) that integrates feature extraction from both Euclidean and feature spaces, enhancing the representation of key geometric features.
3. To validate the performance of the proposed framework and compare it with existing methods under practical farming conditions.

**Figure 1:**
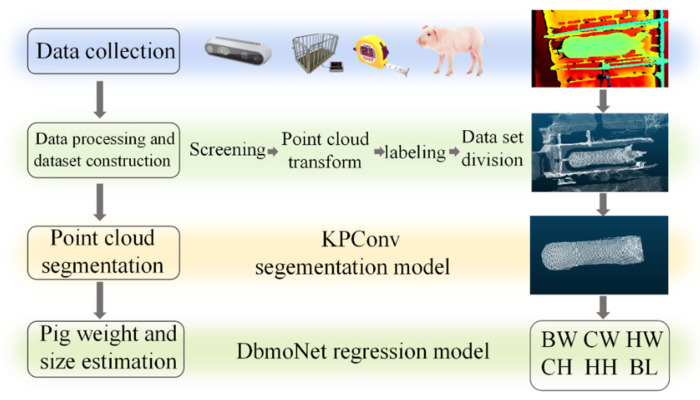
Body weight and size estimation overall architecture: BW: body weight; CW: chest width; HW: hip width; BL: body length; CH: chest height; HH: hip height.

The overall workflow of this framework follows a four-stage pipeline, as illustrated in Figure 1: data collection, data preprocessing and dataset construction, point cloud segmentation, and estimation of body weight and size.

The remainder of this paper is organized accordingly: Section 2 details the data collection and processing pipeline. Section 3 introduces the methodology, including the KPConv segmentation model and the DbmoNet regression model. Section 4 presents the experimental results. Section 5 provides discussion, and Section 6 concludes the study.

## 2. Dataset Construction

### 2.1. Sows and Housing

The experimental data were collected from a commercial sow farm situated in the Yabuli region of Heilongjiang Province, Northeast China. The data collection period was from 19 December 2022 to 15 January 2023, totaling 28 days. The sow building was semi-enclosed with windows, utilizing natural light during the day and artificial lighting (40 W incandescent lamps, ~20–30 lux) in the evening. Individual stalls (2.14 m × 0.7 m × 1.25 m) were used to feed sows. Sixty primiparous and multiparous non-pregnancy sows were selected, including Large White sows, Landrace sows, and cross-breeding sows (Landrace sows × Large White sows) for data collection. The average weight of sows was 211.5 kg. Different sows were identified through ear tags.

### 2.2. Data Collection

Data were acquired continuously daily from 8:00 a.m. to 6:00 p.m. The collected data included body size, body weight, and images of the sows’ backs. An Intel^®^ RealSense™ D455 depth camera (Santa Clara, CA, USA) was used for image acquisition. The color and depth images were set to a resolution of 400 × 240 pixels, with a frame rate of 30 fps. The depth camera’s horizontal and vertical fields of view (FOV) were 87° and 58°, respectively. The depth camera was mounted on a bracket fixed above the sow stall, positioned 210 cm above the scale surface to capture images vertically from above.

**Figure 2:**
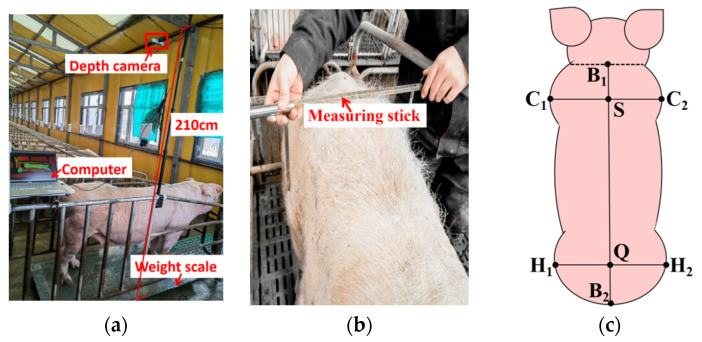
Body weight and size data acquisition: (**a**) body weight and image acquisition equipment; (**b**) body size acquisition process; (**c**) body size feature point.

Weight was measured using a scale with a measurement range from 0 to 800 kg, with a precision of 0.5 kg. During data collection, each sow was driven to a limit bar equipped with a depth camera and a weight scale to obtain weight data and images of the sow’s back. The image and weight data acquisition equipment is shown in Figure 2a. The depth camera was connected to the control computer via a USB cable, and video capture was manually controlled using Intel RealSense Viewer v2.50.0 software.

A tape measure and a measuring rod were used to collect body scale data. The measuring rod had an accuracy of 0.5 cm, the tape measure had a reading accuracy of 0.1 cm, and the overall measurement accuracy for body size was 0.5 cm. Measurements were taken while the sows were standing upright. The measurement process is illustrated in Figure 2b. Measurements were performed by the same person, and the final value was determined as the average of three measurements for each body size. The body size measurements included five dimensions as shown in Figure 2c: chest width (CW), hip width (HW), chest height (CH), hip height (HH), and body length (BL). The widest points C_1_ and C_2_ of the chest were used to measure chest width, with the midpoint S of the line connecting C_1_ and C_2_ serving as the chest height measurement point. The widest points H_1_ and H_2_ of the hip were used to measure hip width, and the midpoint Q of the line connecting H_1_ and H_2_ was used to measure hip height. The midpoint B_1_ of the neck and B_2_ of the tail were used to measure body length.

### 2.3. Data Description

A total of 240 sets of body weight and size data were obtained from 60 sows, with each sow measured multiple times over the 28-day experimental period to ensure data independence and statistical validity. The statistical analysis results are shown in Table 1. The maximum weight of sows is 267 kg, the minimum weight is 167 kg, the average weight is 211.5 kg, and the standard deviation is 20.8 kg. The mean values of CW, HW, BL, CH and HH are 36.4 cm, 36.9 cm, 86.6 cm, 90.1 cm and 84.6 cm, respectively, and the standard deviations are 2.5 cm, 2.5 cm, 2.7 cm, 5.6 cm, and 2.5 cm, respectively.

The considerable variability observed across all parameters (e.g., 20.8 kg for body weight and 5.6 cm for chest height) confirms a diverse representation of sow morphology within the studied population. This diversity is essential for building robust models, as it helps prevent overfitting and supports generalization.

### 2.4. Data Processing

The collected images had to be processed before they could be used. The image processing involved two aspects: image selection and image transformation. Image selection was performed manually to remove incomplete images. All depth image visualization, manual inspection, and preliminary filtering to select frames containing a complete sow body were performed using CloudCompare v2.11.3 software. After the completion of the image selection process, image transformation was required to convert the collected depth images into 3D point clouds. This transformation was based on the camera parameters of the focal lengths *f_x_* = 215.9346 mm and *f_y_* = 215.9346 mm. The data was converted from the image coordinate system to the world coordinate system using these parameters. The calculation formula is shown in Equation (1), in which *x*, *y*, *z* are the point cloud coordinates, *D* is the depth value, *f_x_* and *f_y_* are the camera focal length, and *x*′ and *y*′ are the depth image coordinates. The conversion from depth images to 3D point clouds was implemented through a custom Python script. The script utilized libraries such as NumPy and Open3D to perform the coordinate transformation defined in Equation (1) efficiently. The converted point cloud includes the background and sow body.

**Figure 3:**
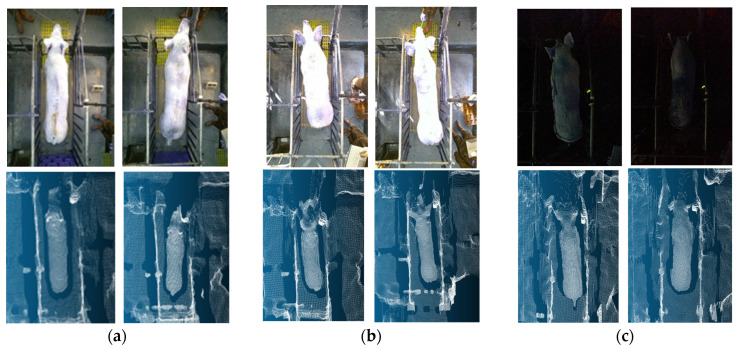
RGB images and corresponding point cloud under different lighting conditions: (**a**) normal; (**b**) strong light; (**c**) low light.

Due to the data being collected at different time periods, there are differences in the brightness of the light. The point clouds, generated from the infrared-based depth sensing mechanism, demonstrate consistent structural integrity across varying visible light conditions, as visually supported by the representative examples in Figure 3. In contrast, the corresponding RGB images exhibit significant variations in brightness.

(1) $$\left[\begin{array}{c} x\\ y\\ z \end{array}\right]=D\left[\begin{array}{ccc} \frac{1}{f_{x}} & 0 & 0\\ 0 & \frac{1}{f_{y}} & 0\\ 0 & 0 & 1 \end{array}\right]\left[\begin{array}{c} x^{\prime }\\ y^{\prime }\\ 1 \end{array}\right]$$

### 2.5. Dataset Division

After screening, the study utilized a total of 12,000 point cloud samples. These samples were derived from 240 sets collected from 60 sows, with four measurements per sow different times. The division of the dataset was performed at the individual sow level to prevent data leakage and ensure complete independence between the datasets. The 60 sows were randomly assigned to the training, validation, and test sets in a ratio of 3:1:1. This resulted in 36 sows (144 datasets, 7200 point cloud samples) for training, 12 sows (48 datasets, 2400 samples) for validation, and 12 sows (48 datasets, 2400 samples) for testing. The 240 sets of body weight and size labels, including body weight (BW), chest width (CW), hip width (HW), body length (BL), chest height (CH), and hip height (HH) were partitioned correspondingly, maintaining a direct match with their respective point clouds, respectively. The dataset division is summarized in Table 2.

## 3. Body Weight and Size Estimation Methods

After completing the dataset preparation, the work was divided into two main parts. The first part involved establishing an automatic segmentation model for sow bodies, which separated the sow body from the background. The second part focused on creating an automatic estimation model for body weight and size. This model utilized the segmented point cloud of the sow’s back as input and generated estimated values for body weight and size.

### 3.1. Segmentation Method for Back Surface Point Cloud

The goal of sow body point cloud segmentation was to separate the sow’s back point cloud from the complex background to eliminate interference. Conventional point cloud segmentation algorithms, including the Euclidean distance algorithm [24], Random Sample Consensus algorithm (RANSAC) [23], and Region Growing method [28], gradually segmented various backgrounds like fences and ground. These algorithms typically involved point cloud computation, resulting in a complex and cumbersome calculation process. To enhance segmentation accuracy and efficiency, automatic point cloud segmentation algorithms were explored. This segmentation process comprised two steps: firstly, labeling the true segmentation values of different points in the point cloud, and secondly, establishing an automatic point cloud segmentation model.

#### 3.1.1. Point Cloud Segmentation Labeling

**Figure 4:**
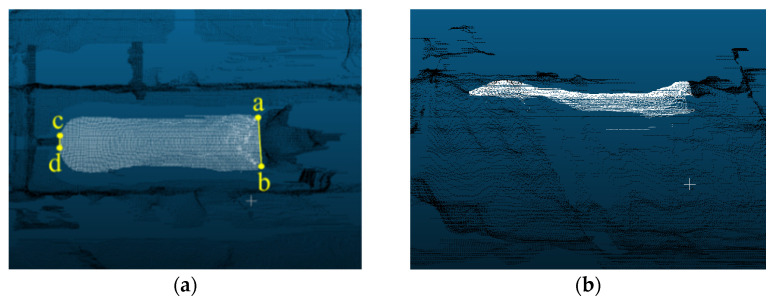
Point cloud labeling: (**a**) top view of the back; (**b**) side view, points a and b are the head and neck segmentation points; points c and d are the tail segmentation points.

The point cloud labeling was performed manually in CloudCompare software. The point cloud was partitioned into two segments: the sow body and the background. To mitigate interference from the head and tail, the back of the sow’s body, excluding the head and tail, was designated as the segmentation target. The feature points for head segmentation are located at the junction of the neck and shoulder, where there is the greatest curvature change, as depicted in Figure 4. Points a and b denote the head and neck segmentation points, respectively, and are connected to delineate the sow’s head point cloud. Similarly, the feature points for tail segmentation are situated where the curvature change in the sow’s buttock tails is most pronounced, with points c and d representing the tail segmentation points. These points are connected to segment the tail. The head, tail, and background of the sow are designated as 0, depicted as black points in Figure 4, while the sow body, with its head and tail removed, is designated as 1, represented by white points. Figure 4a displays the top view of the labeled point cloud, while Figure 4b illustrates the side view.

#### 3.1.2. KPConv Segmentation Model

**Figure 5:**
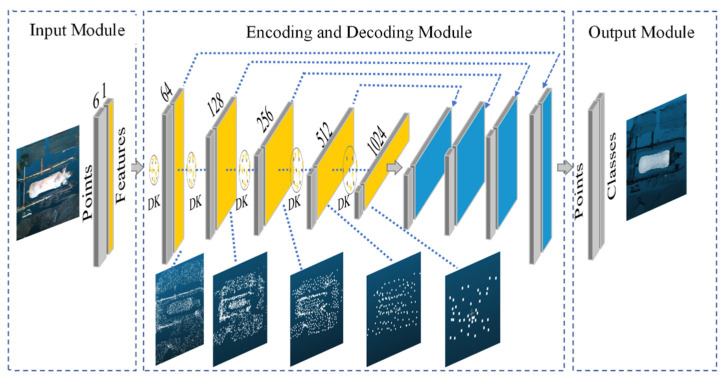
KPConv segmentation model architecture. DK: deformable kernels. Yellow region: feature extraction and downsampling. Blue region: feature propagation and upsampling.

Due to the sow body’s inherent flexibility in motion, a KPConv-based point cloud segmentation model [37] with deformable convolution kernels was developed for sow body samples in various poses. The overall architecture of the KPConv model is illustrated in Figure 5. The model consists of three main components: an input module, an encoder–decoder module, and an output module. The input module is responsible for processing the raw point cloud data of the sow body. The encoder–decoder module performs feature extraction and propagation through two key mechanisms: (1) feature extraction and downsampling, where the number of feature points is reduced layer by layer while the feature dimension per point increases, and (2) feature propagation and upsampling, which integrates nearest neighbor interpolation with layer-wise feature propagation. This hierarchical design allows the model to progressively capture both local geometric details and broader contextual semantic information, which is essential for accurate segmentation of the sow’s body profile. The output module produces the final classification results for the original point. The core mechanism involves kernel point convolution to aggregate neighboring features, as formalized in Equation (2) [37].

(2) $$(F*g)(x)=\sum_{x_{i}\in N_{x}}g_{deform}(x-x_{i},\Delta (x))f_{i}$$

where F denotes the point’s features, g denotes the kernel function, F*g denotes the convolution of the two. *x_i_* is the point *x* within the neighborhood space *N_x_*, *N_x,_* defined by a spherical query with radius *r*, *f_i_* is the feature of the neighborhood point *x_i_*; the function *g* is the kernel point convolution operation, the point convolution kernel used in this model is deformable kernel, and Δ(x) is the offset of the kernel points. For each kernel, the model generates offset vectors to adjust kernel point positions based on local point cloud structures, creating deformable kernels that enhance feature extraction for sow bodies in varying poses.

### 3.2. Model Development of Body Weight and Size Estimation

**Figure 6:**
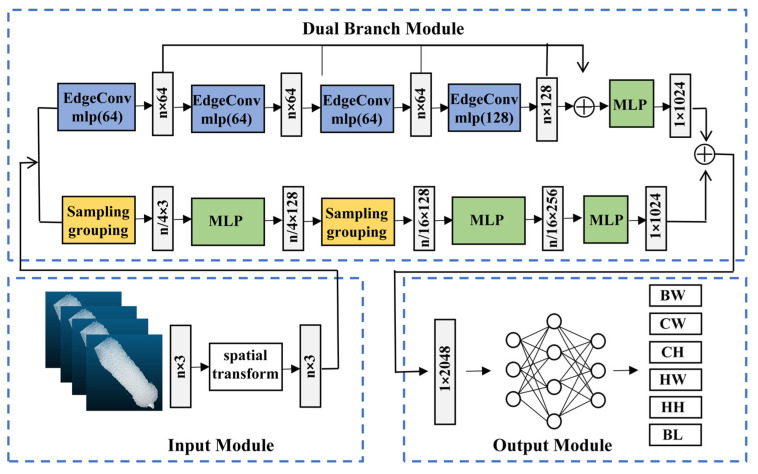
The DbmoNet model for body weight and size estimation. Sampling grouping: uses FPS and multi-scale radius grouping to capture local geometric structures. EdgeConv: dynamically constructing graphs in feature space to aggregate semantic features. MLP: applies shared perceptron layers for feature transformation and aggregation. BW: body weight. CW: chest width. HW: hip width. BL: body length. CH: chest height. HH: hip height.

The segmented back point cloud obtained from the KPConv model serves as the direct input to the DbmoNet regression model for estimating body weight and size. The dual-branch network structure has two parallel branches that extract nearest neighbor features in the distance space and the feature space for the same input. This architecture is designed to jointly model both the stable spatial structure and the semantic similarity among points, which is essential for comprehensively characterizing key geometric features of the sow‘s back. By integrating these complementary perspectives, the model is expected to improve the richness of feature representation and, consequently, the accuracy of estimation. The structure of the proposed DbmoNet model is shown in Figure 6.

It includes an input module, a dual-branch module for feature extraction, and an output module. The input module completes the initial transformation of the point cloud, the dual-branch module is the core module for feature extraction, and the output module integrates the features for the final estimation. A dual-branch module was established for parallel feature extraction. The branch based on location nearest neighbors extracted features from k-nearest neighbors in the distance and had a downsampling process. The branch based on feature nearest neighbors extracted features from k-nearest neighbor points in the feature space without downsampling. Two parallel methods were used to extract complementary features, and the extracted feature dimensions were all 1 × 1024. The features of the two branches were connected at the end of the model.

#### 3.2.1. Point Cloud Spatial Transformation

DbmoNet model took the segmented sow back point cloud as input, randomly sampled *n* points, and the input dimension was *n* × 3. Due to the rotation invariance of the sow point cloud, the input original point cloud was rotated to an angle that was conducive to predicting regression results through the spatial transform module. Specifically, an alignment network was inserted into the input point cloud, and a transformation matrix *θ* was predicted to align different inputs. The *P_i_* coordinate of a point in the origin cloud was (*x_i_*, *y_i_*, *z_i_*), and the transformed point *P_i_*′ coordinate was (*x_i_*′, *y_i_*′, *z_i_*′). The formula for calculation is presented in Equation (3), where *θ* represents the transformation matrix, and (*x_i_*′, *y_i_*′, *z_i_*′) denotes the coordinates after transformation, obtained by multiplying the pre-transformation coordinates (*x_i_*, *y_i_*, *z_i_*) by the transformation matrix *θ*. After transformation, there were still n points with a dimension of *n* × 3, and they were input into the dual-branch module for feature extraction and aggregation.

(3) $$\left[\begin{array}{ccc} x_{i}^{\prime } & y_{i}^{\prime } & z_{i}^{\prime } \end{array}\right]=\left[\begin{array}{ccc} x_{i} & y_{i} & z_{i} \end{array}\right]\left[\begin{array}{ccc} \theta_{00} & \theta_{01} & \theta_{02}\\ \theta_{10} & \theta_{11} & \theta_{12}\\ \theta_{20} & \theta_{21} & \theta_{22} \end{array}\right]$$

#### 3.2.2. Branch Based on Location Nearest Neighbors

The dual-branching module is the main module for feature extraction in the model. In this, the branch based on location nearest neighbors divides the neighboring points on the coordinates into a group for feature extraction. The process of feature learning involved three steps: sampling, grouping, and feature extraction. When the input points {*p*_1_, *p*_2_, …, *p_n_*} of the rotated sow point cloud were given, iterative farthest point sampling (FPS) was adopted to select points {*p_i_*_1_, *p_i_*_2_, …, *p_im_*}, such that *p_ij_* became the farthest point to the set {*p_i_*_1_, *p_i_*_2_, …, *p_ij_* − 1}. Each downsampling reduced the number of points in the previous layer by a factor of 1/4. Multiple Scale Grouping (MSG) was implemented to assign different sampling radii for grouping, and features of the sow point cloud with various radii were combined to create multi-scale features. The feature extraction process entailed extracting features of the point *p_i_* and its *k*-nearest neighbor points through MLP, which comprised Convolution, BatchNorm, Relu, and MaxPool layers. The selection of k-nearest neighbor points was based on the Euclidean distance in the coordinate space of the sow point cloud. For a point *p_i_* with coordinates (*x_i_*, *y_i_*, *z_i_*) and another point *p_j_* with coordinates (*x_j_*, *y_j_*, *z_j_*), their Euclidean distance is computed as shown in Equation (4). The set of *k*-nearest neighbors of point *p_i_* in coordinate space, denoted as *N_k_^coord^*(*p_i_*), is then defined as the set of *k* points *p_j_* ( where *j* ≠ *i*) with the smallest distances *d_coord_*(*p_i_, p_j_*).

(4) $$\;d_{coord}(p_{i},p_{j})=\sqrt{{\left(x_{i}-x_{j}\right)}^{2}+{\left(y_{i}-y_{j}\right)}^{2}+{\left(z_{i}-z_{j}\right)}^{2}}$$

The branch focusing on location nearest neighbors underwent two rounds of sampling, grouping, and feature extraction to generate *n*/16 × 256 feature layers. Subsequently, one MLP step was applied to aggregate the input sow point cloud into 1 × 1024 features.

#### 3.2.3. Branch Based on Feature Nearest Neighbors

When extracting point cloud features from the sow body surface, the point cloud was treated as a graph for feature extraction. The points within the point cloud were regarded as vertices of the graph, and the connections between these vertices were extracted as edges. A point cloud dynamic graph convolutional network was established as a feature extraction branch within a dual-branch structure. The branch based on feature nearest neighbors clustered points with similar features together for subsequent feature extraction. The primary feature extraction module utilized was EdgeConv, which extracted edge features to characterize the relationships between points and their neighboring points. The EdgeConv operation updates the feature *f_i_* of point *p_i_* by aggregating features from its neighbors in the feature space, as defined in Equation (5).

(5) $$f_{i}^{\left(l+1\right)}=\underset{p_{j}\in N_{k}^{feat}(p_{i}^{\left(l\right)})}{\max}h\Theta \left(f_{i}^{\left(l\right)},\;f_{j}^{\left(l\right)}-f_{i}^{\left(l\right)}\right)$$

Here, *f_i_*^(*l*)^ represents the feature of point *p_i_* at layer *l*, and *hΘ* is a learnable function implemented by an MLP. The max operation denotes a channel-wise max pooling layer. The neighborhood point *p_j_* is selected as the *k*-nearest neighbor point of *p_i_* in the feature space. The set *N_k_^feat^*(*p_i_*^(*l*)^) is the set of *k*-nearest neighbors of point *p_i_* in the feature space at layer *l*, determined by the Euclidean distance between point features, as shown in Equation (6).

Among them, the initial value of the feature *f_i_* of the vertex *p_i_* is the position coordinate value of the point *p_i_*. After each EdgeConv module, the vertex *p_i_* would regenerate the feature *f_i_*, and find the *k*-nearest neighbor points in the feature space to reconstruct the feature map. Therefore, in the branch based on feature nearest neighbors, the feature map changed dynamically.

(6) $$d_{feat}(f_{i},f_{j})=∥f_{i}-f_{j}{∥}_{2}$$

Here, *N_k_^feat^*(*p_i_*^(*l*)^) consists of the *k* points *p_j_* with the smallest feature distances *d_feat_*(*f*_*i*_^(*l*)^,*f*_*j*_^(*l*)^) to *p_i_*.

After four iterations of the EdgeConv MLP module, the extracted features from different levels were combined and ultimately aggregated into 1 × 1024 features. Additionally, the features from the location-based feature extraction branch, which were also 1 × 1024, were merged into 1 × 2048 features, completing the feature extraction and aggregation process.

The branch based on feature nearest neighbors excelled at extracting features from points with similar characteristics in the feature space, whereas the branch based on location nearest neighbors was adept at capturing features from neighboring points in the positional space. For instance, all points along the spine of a sow’s back typically share similar height characteristics while aligning in a straight line in positional space. The 1 × 2048 features obtained through the dual-branch structure were better suited to extract and aggregate features from the points along the spine region.

#### 3.2.4. Output Module

The output module of the DbmoNet model primarily utilized a fully connected layer. As depicted in Figure 6, this module received the 1 × 2048 features extracted by the dual-branch feature extraction module. It then conducted feature dimension reduction via the fully connected layer, ultimately producing estimated values for body weight and size, denoted as BW, CW, HW, CH, HH, and BL, respectively.

To assess the efficacy of the proposed DbmoNet model, various output regression estimation models for body weight and size were developed based on the PointNet++, KPConv, and DGCNN classification algorithms for comparative analysis. The final Softmax layer of the PointNet++, KPConv, and DGCNN classification algorithms were eliminated, and the output neurons were adjusted to six, aligning with the output values corresponding to BW, CW, HW, CH, HH, and BL, respectively.

### 3.3. Experiment and Parameter Setting

The model was implemented using Python 3.8.0 and PyTorch 1.13.1 libraries, and the computer was configured with 32GB memory; Windows10(64-bit) system; the CPU was Intel i7-9700 3.0 GHz; the GPU was NVIDIA Tesla T4 with 16GB of independent video memory.

During the body size estimation, the batch size of the training of PointNet++, KPConv, DGCNN and DbmoNet models was 8, the number of epochs was 310, and the models were optimized by Adam. The learning rate was set to 0.01 for PointNet++ and KPConv models, and 0.001 for DGCNN and DbmoNet models.

Mean square error (MSE) was used as the Loss function. The calculation is shown in Equation (7), where *MSE_BW_*, *MSE_CW_*, *MSE_HW_*, *MSE_CH_*, *MSE_HH_* and *MSE_BL_* indicate the MSE corresponding to estimated BW, CW, HW, CH, HH, and BL, respectively. They were summarized as the Loss value, so that the deviation in body size and weight of each dimension had an effect on the final Loss value. Using MSE as the Loss value, the Loss value was more sensitive to the change in deviation after the error was squared, which was conducive to the adjustment of model parameters.

Loss = *MSE_BW_* + *MSE_CW_* + *MSE_HW_* + *MSE_CH_* + *MSE_HH_* + *MSE_BL_* (7)

The calculation of *MSE_k_* for each body size parameter *k* is shown in Equation (8), where *k* ∈ {BW, CW, HW, CH, HH, BL}. Here, *n* denotes the number of training samples. For the *k*-th parameter, *y_i_*^(*k*)^ represents the true value of the *i*-th sample, and *y_i_*′^(*k*)^ denotes the corresponding predicted value.

(8) $$MSE_{k}=\frac{1}{n}\sum_{i=1}^{n}{\left(y_{i}^{\left(k\right)}-y_{i}^{\prime \left(k\right)}\right)}^{2}$$

### 3.4. Evaluation Metrics

#### 3.4.1. Segmentation Models Evaluation Metrics

To evaluate the performance of the segmentation model, several metrics were utilized: overall segmentation accuracy (OA), mean intersection over union (mIoU), precision, recall, and F1 score. OA indicates the ratio of correctly predicted data points to the total data points, while precision measures the accuracy of sow body predictions and recall assesses the proportion of correctly detected sow body data points. F1 score provides a combined assessment of accuracy and recall. Intersection over union (IoU) quantifies the overlap between predicted and true values, where *IoU*_1_ represents the IoU of sow bodies and *IoU*_2_ represents the IoU of the background. Mean intersection over union (mIoU) represents the average IoU across sow bodies and background. Defined in Equations (9)–(14), False Positive (FP) refers to points incorrectly predicted as sow bodies, True Positive (TP) refers to correctly predicted sow bodies, False Negative (FN) refers to points incorrectly predicted as background, and True Negative (TN) refers to correctly predicted background points.

(9) $$Accuracy=\frac{TP+TN}{TP+TN+FP+FN}$$

(10) $$Precision=\frac{TP}{TP+FP}$$

(11) $$Recall=\frac{TP}{TP+FN}$$

(12) $$F1-score=\frac{2Recall\times Precision}{Recall+Precision}$$

(13) $$IoU_{i}=\frac{TP_{i}}{TP_{i}+FP_{i}+FN_{i}}$$

(14) $$mIoU=\frac{1}{2}\left(IoU_{1}+IoU_{2}\right)$$

#### 3.4.2. Body Weight Size Estimation Models Evaluation Metrics

To assess the performance of multiple output regression models, several metrics were utilized: root mean squared error (RMSE), mean absolute error (MAE), total mean square error (MSE), mean absolute percentage error (MAPE), and relative error. RMSE, MAE, and MSE quantify the absolute error between predicted and true values, where smaller values indicate better model performance. MAPE measures the relative error, enabling comparative analysis across models. Relative error assesses the model’s accuracy in estimating individual sample errors. The calculation of MSE is detailed in Equation (8), while RMSE, MAE, MAPE, and relative error are defined in Equations (15)–(18), with n denoting the number of test samples, *y_i_* representing the true value of the *i*-th sample, *y_i_*′ indicating the predicted value, and $\bar{y_{i}}$ representing the average of all true values in the test samples.

(15) $$RMSE=\sqrt{\frac{1}{n}\sum_{i=1}^{n}{\left(y_{i}-y_{i}^{\prime }\right)}^{2}}$$

(16) $$MAE=\frac{1}{n}\sum_{i=1}^{n}\left|y_{i}-y_{i}^{\prime }\right|$$

(17) MAPE=1n∑i=1nyi−yi′yi×100%

(18) Relative error=(yi−yi′)yi

## 4. Results

### 4.1. Sow Body Segmentation

#### 4.1.1. Segmentation Model Training Results

**Figure 7:**
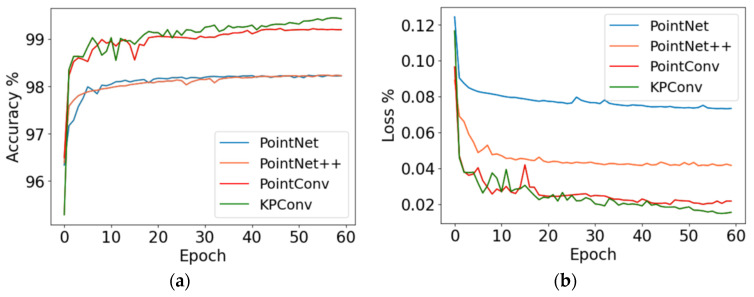
Accuracy and loss during training of models: (**a**) the Accuracy; (**b**) the loss value.

The sow body segmentation model using KPConv was established, with point cloud segmentation algorithms such as PointNet, PointNet++, and PointCNN used for comparison. All comparative models were trained and evaluated under identical conditions using the same input data to ensure a fair performance comparison. The training process of the model is depicted in Figure 7. The KPConv model achieved a final accuracy convergence value of 99.43%, surpassing the PointNet model by 1.21%, the PointNet++ model by 1.20%, and the PointCNN model by 0.23%. The Loss value of the KPConv model is 0.01, which is lower than the other three models by 0.03, 0.02 and 0.01, respectively. Given the high accuracy achieved and to mitigate the risk of overfitting, training was stopped at this stage. All four models have high segmentation accuracy, with the KPConv segmentation model having the best training results.

#### 4.1.2. Segmentation Model Test Results

**Figure 8:**
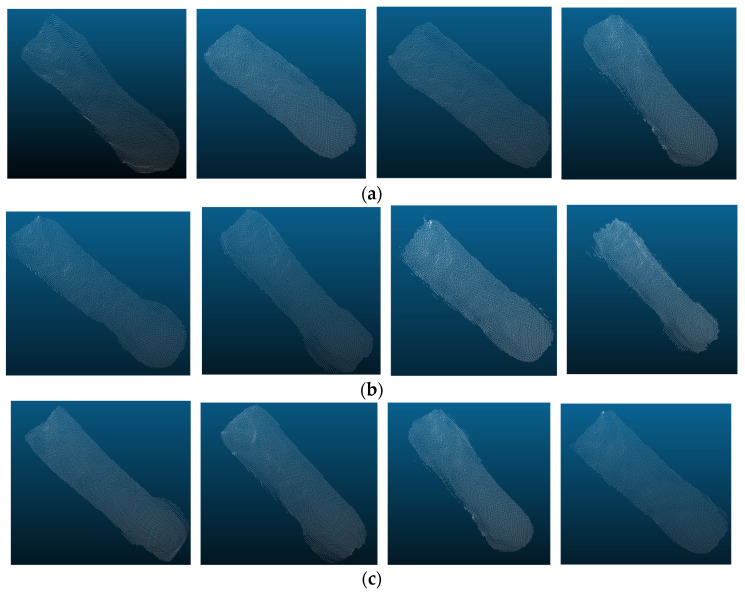
The segmentation effect of partial samples: (**a**) the segmentation boundaries are clear; (**b**) the segmentation boundaries at the neck are unclear; (**c**) the segmentation boundaries at the chest and buttocks are unclear.

As shown in Table 3, the OA, precision, recall, F1 score, and mIoU of the KPConv segmentation model are 99.54%, 97.73%, 97.30%, 97.52% and 97.32%, respectively. It can be seen that KPConv segmentation model has the best segmentation performance with all evaluation metrics values higher than other models. Since the KPConv algorithm uses deformable convolution kernel points, it enables the feature extraction process to adapt better to the input samples and therefore has better segmentation results. Figure 8 shows the segmented sow body point cloud.

Figure 8a depicts samples with good segmentation effect, displaying a clear outline of the sow’s body. Figure 8b illustrates samples with unclear and inaccurate neck segmentation boundaries, possibly due to difficulties in clearly defining the boundaries during manual labeling. Figure 8c demonstrates samples with inaccurate segmentation of the chest and buttocks edges. The width of the chest and buttocks often leads to contact with the side fence, resulting in unclear segmentation boundaries. Overall, the segmented samples effectively represented the body shape of the sows and provided a good basis for the next estimation of body weight and body size.

### 4.2. Body Weight and Size Estimation

#### 4.2.1. Estimation Model Training Results

**Figure 9:**
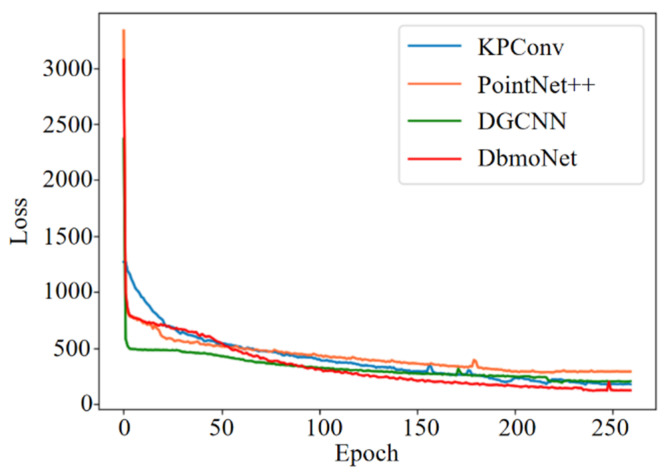
Loss values for the validation set of each model during training.

PointNet++, KPConv, DGCNN and DbmoNet body weight and body size estimation models were trained, respectively. The Loss value of the validation set varies with the number of iterations, as shown in Figure 9. The PointNet++, KPConv, DGCNN and DbmoNet models finally converge to 288.62, 202.24, 175.40 and 121.23, respectively. It can be seen that DbmoNet model has the lowest Loss value, and has the best body weight and size estimation effect compared with other models. Since the DbmoNet model uses a two-layer architecture to extract features, the model size is relatively large, so the convergence speed of the model is intermediate.

#### 4.2.2. Estimation Model Test Results

In terms of the test set, the performance of PointNet++, KPConv, DGCNN and DbmoNet body weight and size estimation models were tested, and the results are shown in Table 4. Among them, the MAE values of the DbmoNet model in BW, CW, HW, BL, CH, and HH are 7.83kg, 1.46cm, 1.24cm, 3.31cm, 1.65cm, and 2.12cm, respectively, while the MAPE values are 3.74%, 3.97%, 3.33%, 3.82%, 1.94%, and 2.43%, respectively. The DbmoNet model has the smallest error and the best performance. The DGCNN and KPConv models have the second-best performance, while the PointNet++ model has the worst performance.

The reason for different performance comes from the ability of model feature extraction. The DbmoNet model has shown the best feature extraction ability in the feature extraction of sow back point cloud, taking into account the nearest neighbors in the feature space and in the spatial position. From Table 1, it can be seen that the average body weight and size values of the sow samples in this study are both relatively large, resulting in a higher final MAE value. However, from MAPE, it can be seen that the percentage of error is within the applicable range.

**Figure 10:**
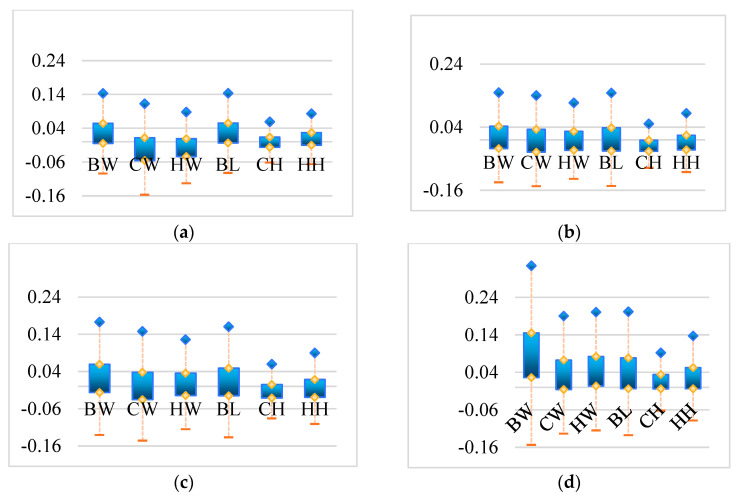
Histogram of relative error for different models regarding BW, CW, HW, BL, CH, HH: (**a**) DbmoNet; (**b**) DGCNN; (**c**) KPConv; (**d**) PointNet++; BW: body weight; CW: chest width; HW: hip width; BL: body length; CH: chest height; HH: hip height.

To more effectively compare the testing outcomes of models on different body weight dimensions, the relative error histograms of the four models for BW, CW, HW, BL, CH and HH were established, as shown in Figure 10.

Comparing the relative error of the four models, it can be seen that the error range of the DbmoNet model in each dimension of BW, CW, HW, BL, CH, and HH is smaller than that of other models. Comparing the BW, CW, HW, BL, CH, HH of each model, the error range of BW is relatively large, while the error ranges of CH and HH are relatively small. The large BW error is due to the difficulty of integrating various aspects of body shape into weight prediction. CH and HH represent the height characteristics of sows, which are the heights from the highest point on the back and the highest point on the buttocks to the ground, respectively. The body size feature point is a single point, making it easier to identify the features of the body size point from the point cloud, resulting in a smaller error range. And features such as width and length need to first identify two feature points, and then establish the relationship between feature points, which is more difficult. Although the target variables differ in their scales, the experimental results indicate that the unweighted loss function yielded balanced predictive performance in this study. The use of a weighted loss remains a valuable direction for future exploration.

## 5. Discussion

### 5.1. Estimation of Body Weight and Size Based on Depth Images

To compare the feature representation capabilities of depth images and point cloud as well as the feature extraction capabilities of 2D and 3D algorithms, the 2D feature extraction algorithm Xception from the literature [31] was used to build a depth image-based model for the estimation of body weight and size. It was tested in comparison with the performance of the 3D feature extraction model DbmoNet, which was built based on point cloud in this study. The results are shown in Table 5. The point cloud-based model works better and is lower than the depth image-based model by 0.19%, 0.12%, 0.06%, 0.58%, 0.09%, and 0.04% on MAPE, respectively.

Two-dimensional feature extraction models based on depth images did not perform very well. One aspect of the reason is that the depth image contains background that can interfere with the estimation results. On the other hand, depth images are still structured 2D images with depth values represented by only 256 quantized values with limited feature representation. Point cloud has better representation of 3D features. Therefore, the point cloud was chosen as the basis for the estimation of body weight and size in this study.

### 5.2. Estimation Results for Different Sow Breeds

The test set of the above model includes all three varieties, and the test results describe the average performance of the mixed samples of the three varieties. Table 6 shows the average values of the body weight and size and the quantity of three varieties in the test samples. It can be seen that the three varieties have different body shape characteristics. The width CW and HW values of Landrace sows are relatively large, and the length value BL is relatively large. The average body weight BW of Large White sows is smaller, with CW, HW, and BL being smaller than the other two breeds. The body size of cross-breeding is between Landrace and Large White sows.

Separate test sets are established for Landrace, Large White, and Cross-breeding sows, and the test results are shown in Table 7. It can be seen that the MAPE values between different breeds are at roughly the same level, and the MAPE values of body weight and length of Cross-breeding sows are smaller. This indicates that the model has the best adaptability to cross-breeding sows, its prediction ability of different breeds is similar, and it has good generalization ability.

Compared with the sows in this study, the Landrace, Large White, and Cross-breeding fattening sows have similar body sizes, but the difference is that the average weight of the fattening sows is smaller and the average weight of the sows is larger. Therefore, in order to apply the model in this article to fattening sows, parameter tuning can be performed through transfer learning to estimate the body weight and size of fattening sows. The proposed method for estimating body weight and size through back point cloud and DbmoNet models can also be applied to other farm animals.

In summary, while the three breeds exhibit distinct morphological characteristics, the model maintains comparable estimation accuracy across them. This indicates that the inherent breed variation within the studied population does not substantially degrade the model’s performance, supporting its robustness for application across these common sow breeds.

### 5.3. Feature Visualization

The interpretable methods for deep learning models currently include Class Activation Maps (CAMs) [40] and Gradient Weighted Class Activation Mapping (Grad CAM) [41], but these methods cannot be applied to algorithms that directly handle point cloud. In order to enhance the interpretability of point cloud processing algorithms and understand from which points the model learns features, inspired by the idea of pixel gradient weighting in Grad-CAM, a feature visualization method of point cloud Gradient Weighted Point Activation Mapping (PAM) is established. The basic idea of PAM is to obtain 11 feature vector of n original points by taking the maximum value method in the layers of feature extraction before feature compression. The size of the feature value of a point can measure its contribution to the final result. The weight is assigned based on the size of the feature value, and the final n points are assigned different weight values. A heat map is used to represent the size of weights.

For the DbmoNet model, the specific steps to generate PAM are as follows:

Step 1: An *n*-dimensional feature vector is obtained. In the dual-branch model, the branch based on feature nearest neighbors retains the positional coordinates of *n* sampled points, while the branch based on location nearest neighbors includes a downsampling process. To obtain the features of *n* points at different levels, the method of nearest neighbor upsampling is used to align the current layer point cloud to the original points. Then, the features of the two branches are connected to obtain the features of *n* points at the current layer.

Step 2: Feature compression is performed. The vector is compressed by the maximum value method and the calculation is shown in Equation (19), where *f_i_* is the feature value of the *i*-th point, *f_ij_* is the *j*-th feature value of the *i*-th point, max is the maximizing function, *i* = 1... *n*, *j* = 1... 384. The *n* × 384 feature vector is compressed to the *n* × 1 feature vector.

(19) $$f_{i}=\max\;f_{ij}$$

Step 3: Weights are assigned to points. The features of n points are normalized to generate weights, as shown in Equation (20), where *ω_i_* represents the weight of the *i*-th point, *i* = 1... *n*. Max is the maximum function and min is the minimum function.

(20) ωi=fi(max fi−min fi)

Step 4: A heat map is generated. Values are assigned to the RGB components of the n original points of the point cloud based on the weights. According to the weight, the value is assigned in the order of R component, G component, and B component. The larger the weight value, the larger the R component assignment and the smaller the B component assignment. The smaller the weight value, the smaller the R component assignment and the larger the B component assignment.

**Figure 11:**
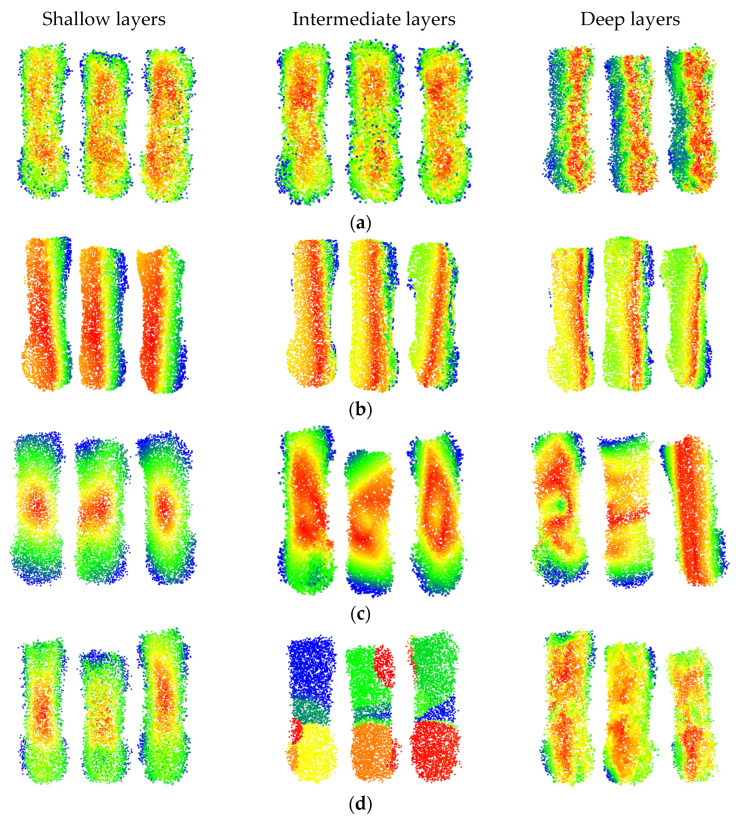
PAM diagram of some test samples: (**a**) DbmoNet; (**b**) DGCNN; (**c**) KPConv; (**d**) PointNet++.

The PAM maps of partial samples at different depths for various models are shown in Figure 11. The red points represent the maximum activation level, the blue points represent the activation level, and points of other colors have intermediate activation levels. The PAM maps of different models at various layers provide a better understanding of the process of feature learning.

It can be observed that the DbmoNet model predominantly learns features of adjacent points in the central region of the body at shallow and intermediate layers, while it learns features of the spine region and one side of the body at deeper layers. From the biological characteristics of sows, points on the spine belong to positions with similar features and adjacent positions, including the length and height characteristics of the sow’s body. One side of the sow body is the nearby points in the position. Due to the roughly symmetrical shape of the sow’s body, width features can be derived from the point cloud on one side. Therefore, there is no need to learn additional body features on the other side, which is more conducive to the rapid convergence of the model.

This effective learning pattern arises from the dual-branch design of DbmoNet. The location-based branch captures stable spatial structures, such as the linear spine and body-side contour, which are fundamental for estimating length, height, and width. Concurrently, the feature-based branch groups points with similar characteristics (e.g., along the curved spine), enabling the model to sensitively perceive continuous shape variations. This complementary mechanism allows the model to fully leverage the body’s approximate symmetry—learning the spine and one side thoroughly provides sufficient information to characterize the entire back accurately and efficiently.

The DGCNN model primarily learns features from points that are similar in nature, and as the depth increases, the learned features gradually tend towards the length direction of the body. Features learned by the KPConv model tend to cover the entire sow’s body as the depth increases, aligning with its principle of continuously extracting features from a larger range of points through point convolution. In contrast, the PointNet++ model learns features of the central region of the sow’s body at shallow layers, gradually extending to features of different areas such as the buttocks, waist, and chest as the model deepens. At deeper levels, the model focuses on features of the central area between the chest and buttocks. Thus, the features learned by the DbmoNet model are more conducive to reflecting the dimensions of the subject, such as length, width, and height.

### 5.4. Comparison to Previous Works

Typical methods of estimating body weight body size in recent years were compared and the results are shown in Table 8.

Compared to previous work, this study has the following advantages:

Firstly, the DbmoNet can achieve simultaneous prediction of body weight and size, including six dimensions: BW, CW, HW, BL, CH, and HH. The training of multi-regression models is more complex than that of single regression models. The Loss value of a multi-regression model needs to be fed back to adjust each of the multiple outputs, thus training the shared network weights. In this study the Loss value is set to the sum of the six output dimensions of MSE, which improves the sensitivity of feedback. Currently, in most research, only body size estimation is generally achieved [11,15,17,22,23,30,36,40], or only body weight estimation is achieved [32,33,34], or body size is estimated first and then body weight is estimated based on body size [8,26,27,28,29].

Secondly, the back point cloud-based method for body weight and body size estimation is not affected by environmental light as compared to the 2D image-based method. The accuracy of the estimation is higher in the point cloud-based method compared to the 3D depth image-based methods. From Figure 2, it can be seen that the sample contains samples under strong and weak light conditions. Even for samples like Figure 2c that cannot be distinguished by the naked eye in RGB images, the integrity of the point cloud is basically not affected. However, the volume measurement estimation method based on 2D RGB images requires image recognition of volume measurement feature points within the visible light range of the naked eye [6]. The MAE values of methods in different studies in Table 8 are not directly comparable due to different experimental samples in different studies. The previous comparison experiments in this study show that the point cloud-based body weight and size estimation model performs better than the depth image-based estimation model.

Thirdly, the method proposed in this study does not involve complex image processing and point cloud processing, and the segmentation of point cloud and the estimation of body weight and size are end-to-end, avoiding the gradual and complex segmentation process of fences, ground, and sow heads and tails in most current studies [8,28]. At the same time, it also avoids the complex process of volumetric feature recognition [27,28,30]. Other methods for estimating body weight and size through deep learning models require preprocessing processes such as resizing [31], cropping [32], and complex point cloud denoising and smoothing [34]. In contrast to the experiment of He et al. [34], the samples in this study were more complete, containing 60 sows of three breeds. Meanwhile, although the initial data collected in the study of He et al. [34] was also point cloud, it was processed as a 2D grayscale image as input to the body weight estimation model, and a 2D convolutional algorithm was used. In contrast, the algorithm used in this study is 3D, which directly processes the point cloud to better preserve the 3D visual features of the sow body.

The subjects of this study are sows in confinement pens, with each image containing only a single animal and no overlap between individuals. Consequently, the direct applicability of this method to free-range conditions with multiple animals is limited and was not evaluated. Acknowledging the sample size of 60 sows, two key considerations arise regarding the model’s wider application. First, for sows, the model’s performance may decline for individuals with body shapes that fall far outside the range captured in our current dataset as its learned features are optimized for the predominant morphology in the study. More fundamentally, the DbmoNet architecture and its effectiveness are intrinsically linked to the specific geometric characteristics of the porcine back. Therefore, direct application to other livestock species with fundamentally different torso topologies and contours is not straightforward and would likely require significant architectural adaptation and retraining. Thus, while this study establishes a robust, non-contact framework for sow body measurement, future work is needed to explicitly test its limits across a wider sow population and to explore transfer learning strategies for adapting the approach to other species.

The automated, non-contact monitoring system presented in this study is expected to generate significant economic benefits for intensive sow production. First, by replacing manual weighing and measuring, it substantially reduces labor costs and minimizes stress-induced productivity losses. Second, the continuous, high-frequency data on body weight and size enable precise feeding strategies and early detection of health issues, potentially improving feed conversion efficiency and reducing veterinary costs. Overall, this technology provides a tool for data-driven management decisions that can enhance both the economic sustainability and animal welfare standards of modern pig farms.

## 6. Conclusions

The following conclusions can be drawn in this study:

1. The KPConv model demonstrated superior effectiveness in segmenting the sow’s back from the background, outperforming other prevalent point cloud segmentation networks and providing a reliable foundation for subsequent biometric analysis.
2. The proposed dual-branch DbmoNet architecture, which explicitly integrates feature space and position space reasoning, achieved the highest overall accuracy in multi-parameter estimation, confirming the value of complementary feature learning for this task.
3. The comparison underscores the superiority of the 3D point cloud paradigm over 2D image-based methods in this context, as directly modeling the three-dimensional surface leads to more robust feature extraction and higher estimation fidelity.
4. From the proposed PAM point cloud feature visualization method, it can be seen that the DbmoNet model mainly learns the key geometric features of the sow spine with similar features and adjacent positions, indicating that the model has the ability to learn effective features.
5. Future research could apply the method to other livestock by optimizing the model, enabling dynamic, multi-species monitoring of body conditions for feeding management and welfare assessment in precise livestock farming (PLF) scenarios.

## Figures and Tables

**Table 1:** Statistical analysis of body weight and size data.

Statistics	BW (kg)	CW (cm)	HW (cm)	BL (cm)	CH (cm)	HH (cm)
Maximum	267.0	41.5	43.0	94.1	111.0	90.0
Minimum	167.0	26.5	30.5	79.5	74.0	77.5
Mean ± Std	211.5 ± 20.8	36.4 ± 2.5	36.9 ± 2.5	86.6 ± 2.7	90.1 ± 5.6	84.6 ± 2.5

Note: BW is body weight; CW is chest width; HW is hip width; BL is body length; CH is chest height; HH is hip height.

**Table 2:** Dataset division.

Data	Training Set	Validation Set	Test Set
Point cloud	7200	2400	2400
Body weight and size	144	48	48

**Table 3:** Test results of segmentation models.

Model	OA (%)	Precision (%)	Recall (%)	F1-Score (%)	mIoU (%)
PointNet	99.22	96.34	94.96	95.65	95.32
PointNet++	99.29	96.89	95.80	96.34	96.07
PointCNN	99.31	96.26	97.13	96.69	96.42
KPConv	99.54	97.73	97.30	97.52	97.32

**Table 4:** Performance comparison of models on test set.

Items	Evaluation Metrics	PointNet++	KPConv	DGCNN	DbmoNet
BW	RMSE (kg)	14.93	12.25	10.59	10.61
	MAPE	6.06%	5.02%	4.12%	3.74%
	MAE (kg)	12.28	10.49	8.41	7.83
CW	RMSE (cm)	1.97	1.95	1.93	1.81
	MAPE	4.52%	4.28%	4.21%	3.97%
	MAE (cm)	1.62	1.58	1.56	1.46
HW	RMSE (cm)	1.61	1.70	1.64	1.58
	MAPE	3.53%	3.75%	3.64%	3.33%
	MAE (cm)	1.31	1.39	1.36	1.24
BL	RMSE (cm)	5.52	4.78	4.41	4.11
	MAPE	5.25%	4.31%	4.13%	3.82%
	MAE (cm)	4.52	3.90	3.59	3.31
CH	RMSE (cm)	2.10	2.42	1.96	2.04
	MAPE	2.04%	2.37%	1.89%	1.94%
	MAE (cm)	1.73	1.99	1.60	1.65
HH	RMSE (cm)	2.55	2.58	2.38	2.82
	MAPE	2.56%	2.55%	2.31%	2.43%
	MAE (cm)	2.20	2.26	1.99	2.12
	MSE (kg^2^)	291.39	219.17	161.95	151.80

Note: BW is body weight; CW is chest width; HW is hip width; BL is body length; CH is chest height; HH is hip height.

**Table 5:** MAPE for depth image-based model and point cloud-based model testing.

Model	BW	CW	HW	BL	CH	HH
Based on depth image	3.93%	4.09%	3.39%	4.40%	2.03%	2.47%
Based on point cloud	3.74%	3.97%	3.33%	3.82%	1.94%	2.43%

**Table 6:** The average value of the body weight and size of different breeds in the test set.

Breeds	Number of Point Cloud Test Sample	BW (kg)	CW (cm)	HW (cm)	BL (cm)	CH (cm)	HH (cm)
Landrace	440	217.44	36.70	37.30	126.92	84.06	86.15
Large White	1400	208.64	36.44	36.55	104.54	84.53	86.53
Cross-breeding	560	213.25	36.43	37.37	122.04	85.35	87.26

**Table 7:** MAPE for different breeds.

Breeds	BW (%)	CW (%)	HW (%)	BL (%)	CH (%)	HH (%)
Landrace	4.00	3.63	3.73	3.97	2.06	2.21
Large White	4.24	4.22	3.69	4.51	1.84	2.23
Cross-breeding	3.40	4.95	3.82	2.98	2.06	2.65

**Table 8:** Comparison of this study to the existing estimation methods.

Automatic Feature Extraction	Images	Studies	Purpose	Methods
No	2D	[6,7]	Body weight	Image processing and machine learning
	3D depth image	[15]	Body size	Image processing
		[8,26,27]	Body weight	Image processing and machine learning
	3D Point cloud	[11,17,22,23,28,29,36,40]	Body size	point cloud processing
			Body weight	point cloud processing and Machine learning
Yes	3D depth image	[31,32,33,42]	body weight	Deep learning
			Body size and body weight	Deep learning
	3D Point cloud	[31,34]	Body weight	Deep learning
Yes	3D Point cloud	This paper	Body size and body weight	Deep learning

## Data Availability

The original contributions presented in this study are included in the article. Further inquiries can be directed to the corresponding author.
